# Metabolic reprogramming mediates hippocampal microglial M1 polarization in response to surgical trauma causing perioperative neurocognitive disorders

**DOI:** 10.1186/s12974-021-02318-5

**Published:** 2021-11-13

**Authors:** Gang Luo, Xiaofeng Wang, Yongchen Cui, Yue Cao, Zhe Zhao, Junfeng Zhang

**Affiliations:** 1 Department of Anesthesiology, Shanghai Sixth People’s Hospital Affiliated to Shanghai Jiaotong University, 600 Yishan Road, Shanghai, 200233 China; 2 Department of Geriatrics, Shanghai Sixth People’s Hospital Affiliated to Shanghai Jiaotong University, 600 Yishan Road, Shanghai, 200233 China

**Keywords:** Microglial polarization, Neuroinflammation, Metabolic reprogramming, Perioperative neurocognitive disorders (PNDs)

## Abstract

**Background:**

Microglial polarization toward pro-inflammatory M1 phenotype are major contributors to the development of perioperative neurocognitive disorders (PNDs). Metabolic reprogramming plays an important role in regulating microglial polarization. We therefore hypothesized that surgical trauma can activate microglial M1 polarization by metabolic reprogramming to induce hippocampal neuroinflammation and subsequent postoperative cognitive impairment.

**Methods:**

We used aged mice to establish a model of PNDs, and investigated whether surgical trauma induced metabolic reprograming in hippocampus using PET/CT and GC/TOF–MS based metabolomic analysis. We then determined the effect of the glycolytic inhibitor 2-deoxy-d-glucose (2-DG) on hippocampal microglial M1 polarization, neuroinflammation, and cognitive function at 3 d after surgery.

**Results:**

We found that surgery group had less context-related freezing time than either control or anesthesia group (*P* < 0.05) without significant difference in tone-related freezing time (*P* > 0.05). The level of Iba-1 fluorescence intensity in hippocampus were significantly increased in surgery group than that in control group (*P* < 0.05) accompanied by activated morphological changes of microglia and increased expression of iNOS/CD86 (M1 marker) in enriched microglia from hippocampus (*P* < 0.05). PET/CT and metabolomics analysis indicated that surgical trauma provoked the metabolic reprogramming from oxidative phosphorylation to glycolysis in hippocampus. Inhibition of glycolysis by 2-DG significantly alleviated the surgical trauma induced increase of M1 (CD86^+^CD206^−^) phenotype in enriched microglia from hippocampus and up-regulation of pro-inflammatory mediators (IL-1β and IL-6) expression in hippocampus. Furthermore, glycolytic inhibition by 2-DG ameliorated the hippocampus dependent cognitive deficit caused by surgical trauma.

**Conclusions:**

Metabolic reprogramming is crucial for regulating hippocampal microglial M1 polarization and neuroinflammation in PNDs. Manipulating microglial metabolism might provide a valuable therapeutic strategy for treating PNDs.

## Highlights

- Surgical trauma impaired the hippocampus-dependent contextual memory in age mice.
- Surgical trauma induced microglial M1 polarization and subsequent neuroinflammation.
- Metabolic reprogramming was crucial for regulating microglial M1 polarization in PNDs.
- Modulating microglial metabolism might provide a valuable approach for treating PNDs.

## Introduction

Perioperative neurocognitive disorders (PNDs) are very common complications after routine surgical procedures, particularly in the elderly [1]. With a steady increase in the surgical geriatric population, PNDs are rapidly becoming a major global health burden [2]. A recent cohort study of older patients undergoing major elective surgery shows that PNDs such as incident delirium and severe delirium are associated with a substantial cost to health care system [3]. The incidence of PNDs ranges from 41–75% at 7 days to 18–45% at 3 months after surgery [1]. PNDs are characterized by mental disorder, memory impairment, and cognitive impairment, which can lead to poor rehabilitation and increased mortality after major surgery [4]. Although various pathological events have been reported to be related to PNDs, accumulating evidence implies that microglial activation and subsequent neuroinflammation are major contributors to the development of PNDs [5–8]. However, the mechanisms underlying microglial activation after surgery are largely elusive.

Immune cells react to their environment by flexibly reprogramming intracellular metabolic pathways that subsequently alter immune function, in a process called immunometabolism [9]. Microglia, the resident immune cells in the central nervous system (CNS), can be activated in a polarizing manner, which is thought to be along a continuum from a classical M1 phenotype to an alternative M2 phenotype under pathological stimulation [10, 11]. The M1 phenotype are capable of producing various pro-inflammatory cytokines, including IL-1β, IL-6, and expressing cell-surface markers such as inducible NO synthase (iNOS) and CD86, whereas M2 phenotype are anti-inflammatory with expressing CD206 and Arginase-1 (Arg-1) [12]. New studying approaches now have unveiled previously unappreciated roles for immunometabolism in regulating microglial polarization and thereby shaping neuroinflammation [13]. Emerging evidence suggests that microglial activation toward a classically pro-inflammatory M1 phenotype is associated with a metabolic reprogramming from oxidative phosphorylation to glycolysis [10, 14, 15]. Indeed, microglial polarization toward M1 phenotype and increased inflammatory cytokines are deeply involved in the cognitive impairments in various neurodegenerative disorders including Alzheimer’s disease and multiple sclerosis [16–18]. Moreover, it has been reported that surgical trauma can shift the dynamic equilibrium of microglial activation toward M1 polarization, exacerbate the neuroinflammation, and induce consequent cognitive impairment in aged mice [19, 20]. The 2-deoxy-d-glucose (2-DG), as a glucose analog, is phosphorylated by hexokinase and thereby competitively inhibits the production of glucose-6-phosphate from glucose and ultimately inhibits glycolysis [21]. Cheng et al. recently demonstrated that early glycolytic reprogramming regulates microglial inflammatory activation, and the inhibition of glycolysis by 2-DG can ameliorate microglial activation-related neuroinflammation and reduce dopaminergic cell death in the Parkinson’s disease animal model [15]. However, the effect of metabolic reprogramming on microglial polarization in response to surgical intervention remains unclear. Furthermore, whether the inhibition of glycolysis by 2-DG could mitigate the surgical trauma induced microglial polarization and postoperative cognitive deficit has not yet been investigated.

Therefore, we hypothesized that surgical trauma can activate microglia toward M1 polarization by metabolic reprogramming to induce neuroinflammation in hippocampus, which ultimately contributes to postoperative cognitive impairment in aged mice. In the present study, we used aged mice to establish a model of PNDs, and investigated whether surgical trauma could induce metabolic reprograming in hippocampus using the 18F-fluorodeoxyglucose (18F-FDG) positron emission tomography/computed tomography (PET/CT) and gas chromatography time-of-flight mass spectrometry (GC/TOF–MS) based metabolomic analysis. We then examined whether inhibition of metabolic reprogramming by glycolytic inhibitor 2-DG could suppress the microglial M1 polarization and neuroinflammation in hippocampus, and thereby improve postoperative cognitive outcomes.

## Methods and materials

### Animals

Male C57BL/6 mice aged 18 months (25–35 g) were obtained from the Shanghai Slac Laboratory Animal Co.Ltd. All experimental procedures were carried out in accordance with Guidelines for Care and Use of Laboratory Animals stipulated by the Animal Ethics Committee of Shanghai Sixth People's Hospital affiliated to Shanghai Jiaotong University. The mice were housed in a temperature-controlled room on a 12-h light/dark cycle with available food and water ad libitum.

### Anesthesia and surgery

We performed the right carotid artery exposure surgery as previously described [22, 23]. Briefly, mice were anesthetized by 2.5% sevoflurane delivered with anesthetic-specific vaporizer. The anesthetic concentrations were monitored with a DATex-Ohmeda monitor. During the procedure, the mouse was kept spontaneous respiration. A 1.5-cm midline neck incision was made after the mouse was exposed to volatile anesthetics at least for 15 min. The soft tissues over the trachea were retracted gently. One centimeter of right common carotid artery was dissected free from adjacent tissues without damaging vagus nerve. The wound was then irrigated and closed using surgical suture. The surgical procedure was performed under sterile conditions and lasted about 15 min. No response to toe pinching was observed throughout the anesthetic and surgical process. Rectal temperature was monitored and maintained at 37 °C with the servo-controlled warming blanket (TCAT-2LV, Physitemp instruments, Clifton, NJ, USA). MouseOX Murine Plus Oximeter System (Starr Life Sciences Corporation, Oakmont, PA, USA) was used to continuously monitor animal’s heart rate and pulse oxygen saturation. After the surgery, all animals received a subcutaneous injection of 0.003 mg/kg bupivacaine. Animals in the control group did not receive anesthesia, surgery or bupivacaine.

### 2-DG administration

2-DG (Sigma-Aldrich, St Louise, USA, F5006) was used as a glycolytic inhibitor for the interventional study. 2-DG was freshly dissolved in sterile normal saline and injected intraperitoneally (i.p.) with 250 mg/kg/day at 30 min before surgery and then once daily for subsequent 2 consecutive days. Vehicle-treated mice received i.p. injections of equal volume of sterile normal saline. The dose of 2-DG was chosen according to Leiter et al.’s study [24].

The mice were randomly assigned to the control, surgery, and 2-DG plus surgery groups. The researcher performing the 2-DG treatment knew the randomized allocation, but the personnel who performed the following assessment, such as behavioral test, et al. were blinded to the randomized allocation and intervention.

### Behavioral tests

All behavioral experiments were conducted between 8:00 am and 5:00 pm in the light phase and recorded by an investigator who was blinded to the animal’s grouping. Between each testing session, the apparatus was cleaned with 75% alcohol to avoid the interference of olfactory cues. 

### Open field test

The exploratory locomotor activities of the mice were evaluated using the open field test at 2 d after surgery. Each mouse was placed in the center of a white opaque plastic chamber (30 × 30 × 40 cm) and allowed to freely explore for 5 min. The total distance of movement and number of center crossings were analyzed using an automated video tracking system (ACT-300A; Coulbourn, USA).

### Fear conditioning test

A fear conditioning test was conducted after surgery as previously described [22, 23, 25]. The training session was conducted at 2 d after surgery. Each mouse was allowed to adapt to the conditioning chamber for 120 s, followed by exposure to an auditory tone (3.6 kHz, 70 dB) and then given an electric foot shock (2 s, 0.8 mA) during the last 2 s of the sound stimulus. Each of these sequences was repeated 3 times, separated by 60 s. When the final foot shock was finished, the mouse stayed in the chamber for another 60 s and then returned to home cage. Twenty-four hours later, the contextual fear conditioning test was performed. Each mouse was placed into the same chamber in which they were trained, observed for 6 min without tone or foot shock, and scored for the freezing behavior. The auditory-cued fear test was performed 2 h after the contextual fear conditioning test in a novel chamber. Each mouse was placed into the novel chamber and allowed to explore for 3 min. Then the training tone was delivered for additional 3 min and the freezing behavior was recorded. Freezing behavior was defined as the absence of all visible movement except for respiration, and it was analyzed and expressed as the percentage of the observation period using an automated video-tracking system (ACT-100A; Coulbourn, USA).

### Immunohistochemistry

As we have described [7], paraffin-embedded hippocampal coronal sections at 5 μm thickness were mounted on Superfrost plus microscope slides. Antigen retrieval with Tris/EDTA buffer (10 mM Tris Base, 1 mM EDTA, 0.05%Tween 20, pH 9.0) was performed at 95–100 °C for 20 min. After being washed in Tris-buffered saline (TBS) containing 0.025% triton-X 100, sections were blocked in 10% donkey serum plus 1% bovine serum albumin in TBS for 2 h at room temperature and then incubated at 4 °C overnight with the following primary antibodies: rabbit polyclonal anti-ionized calcium binding adapter molecule 1 (Iba-1) antibody (1:500, 019–19,741, Wako Chemicals, USA). The sections were incubated with the donkey anti-rabbit IgG antibody conjugated with NL557 (1:200, Catalog Number: NL004, R&D Systems, Minneapolis, MN, USA) for 1 h at room temperature in the dark, and then counterstained with Hoechst 33,342 (1:5000, Catalog Number: 62249, Thermo Fisher Scientific, Waltham, MA, USA) for nuclear staining. After washed in TBS, the sections were mounted and coverslipped with Permount (Catalog Number: 5027797, Fischer Scientific, Waltham, MA, USA). Images were captured by a fluorescent microscope (Leica DM IL LED, Buffalo Grove, USA). A negative control without incubation with primary antibody was performed in all experiments. The quantification was performed as described previously [22]. Briefly, three independent microscopic fields in each section were randomly acquired in the hippocampal CA3 or dentate gyrus area using a counting frame size of 0.4 mm^2^. Three sections per mouse were imaged. The number of pixels per image with intensity above a predetermined threshold level was considered as positive stained areas and quantified by Image J (National Institutes of Health, Bethesda, USA). The level of positive immunoreactivity was reflected by the percentage of the positively stained area in the total area of the image. All quantitative analyses were performed in a blinded fashion.

### Isolation of microglia by Percoll density gradient centrifugation

Microglia were isolated from hippocampal homogenates as described previously [26, 27]. In brief, hippocampal tissue was homogenized in HBSS (pH 7.4) by passing through a 70 μm nylon cell strainer. Resultant homogenates were centrifuged at 600×*g* for 6 min. Supernatant was removed and resulting pellet was re-suspended in 70% isotonic Percoll (GEHealthcare, Pittsburgh, PA, USA) at room temperature. A discontinuous Percoll density gradient was layered as follows: 70%, 50%, 35%, and 0% isotonic Percoll. The gradient was centrifuged for 20 min at 2000×*g* and microglia were collected from the interphase between 70 and 50% Percoll layers [27]. Cells were washed and re-suspended in sterile HBSS. Viable cells were counted by 0.1% trypan blue staining using a hemacytometer. These cells were referred to as enriched microglia on the basis of previous studies showing that viable cells isolated through Percoll density gradient produces greater than 90% microglia [26, 27].

### Measurements of microglial phenotype markers using real-time PCR

M1(iNOS/CD86) and M2(Arg-1/CD206) microglial phenotype markers in enriched microglia by Percoll density gradient centrifugation from hippocampus were measured using real-time PCR [27]. RNA was isolated using the RNeasy plus mini kit (Qiagen, Valencia, CA, USA). RNA concentration was determined by spectrophotometry (Eppendorf, Hauppauge, NY, USA), and RNA was reverse transcribed to cDNA using an RT-RETROscript kit (Ambion, Austin, TX, USA). Quantitative PCR for iNOS, CD86, CD206, and Arg-1 genes was performed using TaqMan probes (Applied Biosystems, Catalog Number: 4316034, ThermoFisher Scientific, Waltham, MA, USA). The mRNA expression was normalized with the reference gene (β-actin). Fluorescence was determined on an ABI Prism 7300-sequence detection system (Applied Biosystems, CA, USA). Data were analyzed using the comparative threshold cycle and the results were represented as fold difference from control.

### PET/CT scan of cerebral glucose metabolism

Mice were scanned to analyze the cerebral glucose metabolism at 3 d after surgery using PET/CT [24]. The mice fasted for 12 h before the examination to avoid the effect of blood glucose on the metabolic distribution of 18F-FDG in brain. Mice received injection of 18F-FDG into tail vein for 30 min. After anesthetized with 2.5% sevoflurane in 100% oxygen, mice were placed in prone position on a scanning board to perform PET/CT scan with Siemens Inveon MicroPET-CT (Siemens Healthcare, Erlangen, Germany). The respiration frequency was monitored and the body temperature was maintained at 37 °C with the servo-controlled warming blanket during the procedure. The scanning field contains the whole brain and neck in mice using 3D model acquisition. Image filter back projection reconstruction in mice axial surface, sagittal and coronal CT images were analyzed. PET-CT image fusion and quantitative analysis of 18F-FDG uptake rate in hippocampal region were acquired by PMOD software (PMOD Technologies, Zurich, Switzerland). Standard uptake value (SUV) in unit of kBq/ml reflects the uptake value per unit volume, which directly reflects the color intensity in the images of PET/CT, but its value is affected by the injection dose per unit body weight and isn’t appropriately used to be compared statistically, so the PET/CT imaging data of SUV were reported using an approach standardized to body weight (bw, in g): SUVbwg/ml = tissue activity (kBq/ml)/injected activity per bw (kBq/g) as previously described [28, 29].

### GC/TOF–MS based metabolomic analysis

The GC/TOF–MS based metabolomics approach was employed to investigate the metabolic profiles in hippocampal tissues harvested at 3 d after surgery using an Agilent 7890B gas chromatograph system (Agilent Technologies, Santa Clara, USA) coupled with a Leco Pegasus HT time-of-flight mass spectrometry as described previously [30]. Metabolites were separated by an DB-5MS column with helium as the carrier gas at a constant flow rate of 1.5 mL/min. The GC/TOF–MS peak spectra were converted into a NetCDF format using ChromaTOF software (version 4.22, Leco, St. Joseph, USA) and directly input into online software and processed by custom scripts in MATLAB (The MathWorks, Inc., Natick, MA, USA). Baseline correction, peak discrimination and alignment, internal standard exclusion, and normalization to the total sum of the spectral intensities with all of the featured metabolites in each sample were conducted. Identification of metabolites was carried out by matching the mass fragments of the differentially expressed metabolites with those of standard substances in commercially available databases, including NIST, NBS, and a self-established library, with 70% similarity threshold. Multivariate analyses were carried out with SIMCA software (version 14.1, Umetrics, Umea, Sweden) as previously described [31]. Principal component analysis (PCA) was processed for checking outlier samples, and orthogonal partial least squares discriminant analysis (OPLS-DA) were processed for identification of differentially expressed compounds. The robustness of OPLS-DA model was validated using the *Q*^2^cum (goodness of the prediction), the *R*^2^Ycum (goodness of the fit) values, and the permutation test (evaluation of the risk of over-fitting). A threshold of 0.5 for *Q*^2^ and *R*^2^Y is widely accepted in model classification to identify good predictive capabilities [32]. The validation plots for OPLS-DA model with permutation tests show correlation coefficients between the original *Y* and the permuted *Y* versus the cum*R*^2^*Y* and *Q*^2^. Fitted regression lines were also displayed, which connects the observed *Q*^2^ to the centroid of permuted *Q*^2^ cluster. The model was considered valid if (1) all *Q*^2^ values from the permuted data set to the left are lower than the *Q*^2^ value on the actual data set to the right and (2) the regression line has a negative value of intercept on the *y*-axis [32, 33]. The significantly discriminant metabolites in t-test (*P* < 0.05) with a variable importance in projection (VIP) values greater than 1.0 in OPLS-DA model was thought to be variables that contributed to differentiation between the two groups. Their structures were then identified by analysis of mass-to-charge ratio m/z of mass spectrometry or precise molecular mass by searching online database (Metlin: http://metlin.scripps.edu). For the metabolic pathway analysis, Kyoto Encyclopedia of Genes and Genomes (KEGG) database were used as reference metabolic pathways.

### Pyruvate and lactate assays

The levels of pyruvate and lactate in samples of hippocampal tissue and enriched microglia from hippocampus were measured as described previously [34] using Pyruvate Assay Kit (A081, Nanjing Jiangcheng Bioengineering Institute, China) and Lactate Assay Kit (A019-2, Nanjing Jiangcheng Bioengineering Institute, China) according to the manufacturer’s instructions, respectively. Lactate/pyruvate ratio (L/P ratio) was calculated to assess the proportion of glycolytic metabolism in relation to mitochondrial oxidative metabolism.

### Western blot analysis

The samples of hippocampal tissue were homogenized in RIPA lysis buffer (Beyotime, P0013C, Shanghai, China) containing freshly added phosphatase and protease inhibitors. Protein concentrations were determined using the BCA Protein Assay Kit (Boster, AR0197, Wuhan, China). 30 μg of protein per lane was separated by 10% SDS-PAGE gels and then transferred to polyvinylidene fluoride membranes (Millipore, Billerica, USA). The blots were blocked in blocking buffer for 2 h at room temperature and then incubated overnight at 4 °C with the following primary antibody: rabbit polyclonal anti-interleukin (IL)-6 antibody (1:1000, Catalog Number:ab6672, Abcam, Cambridge, USA), rabbit polyclonal anti-interleukin (IL)-1β antibody (1:1000, Catalog Number: ab15077, Abcam, Cambridge, USA), and rabbit polyclonal anti-glyceraldehyde 3-phosphate dehydrogenase (GAPDH) antibody (1:5000, Catalog Number: G9545, Sigma Aldrich, St. Louis, MO, USA). After incubation with horseradish peroxidase (HRP)-conjugated goat anti-rabbit secondary antibodies (1:2000, Boster, Wuhan, China) for 2 h at room temperature. The enhanced chemiluminescence (ECL) detection reagents (Thermo Scientific, Rockford, USA) was used to visualize the protein bands. The density of protein band was detected by image analysis system (Image Quant Ai600, General Electric Co., USA), and the relative protein expression of interleukin-6 (IL-6) and interleukin-1β (IL-1β) was normalized to GAPDH.

### Microglial staining and flow cytometry

To study the polarization phenotype of activated microglia in hippocampus after surgery, flow cytometry was performed on enriched microglia from hippocampal tissue. The microglial surface antigens were stained as previously described [27]. In brief, the single cell suspensions were blocked with anti-CD16/CD32 antibody (1:300, eBioscience, San Diego, CA, USA) to prevent Fc receptors binding. Cells were washed and then incubated with the following appropriate primary antibodies: CD45 (1:100, 30-F11, eBioscience, San Diego, CA, USA), CD11b (1:100, M1/70, eBioscience, San Diego, CA, USA), CD86 (1:100, GL1, eBioscience, San Diego, CA, USA), and CD206 (1:100, MR6F3, eBioscience, San Diego, CA, USA) antibodies for 45 min at room temperature. Cells were washed and then re-suspended in FACS buffer (2% FBS in HBSS with 1 mg/ml sodium azide) for analysis. Isotype-matched control antibodies were used as negative controls. Gating was determined based on appropriate negative isotype stained controls. A four-laser Becton–Dickinson FACS Calibur flow cytometer (BD Biosciences, San Jose, CA, USA) was used to collect the data and FlowJo software 10 (FlowJo LLC, Ashland, USA) was used for analysis. Microglia were identified as CD45^+^CD11b^+^ cells. Polarization states of CD45^+^CD11b^+^ microglia were then evaluated according to the surface expression of M1 marker CD86 and M2 marker CD206. M1 microglia was defined as CD86^+^CD206^−^ and M2 microglia was defined as CD86^−^CD206^+^.

### Statistical analysis

Statistical analyses were performed with the GraphPad Prism software (version 8.3, San Diego, CA, USA). The Shapiro–Wilk normality test was used to evaluate data distribution. The analysis showed normal distribution for all continuous variables in present study. Thus, all continuous variables were expressed as mean ± standard deviation (SD). The multiple comparisons were assessed by one-way ANOVA followed by a Tukey post hoc test. Differences between two groups were analyzed with Student's t test. A *P* value < 0.05 was considered to be statistically significant.

## Results

### Surgery trauma, but not anesthesia, induced postoperative cognitive dysfunction in aged mice

**Fig. 1:**
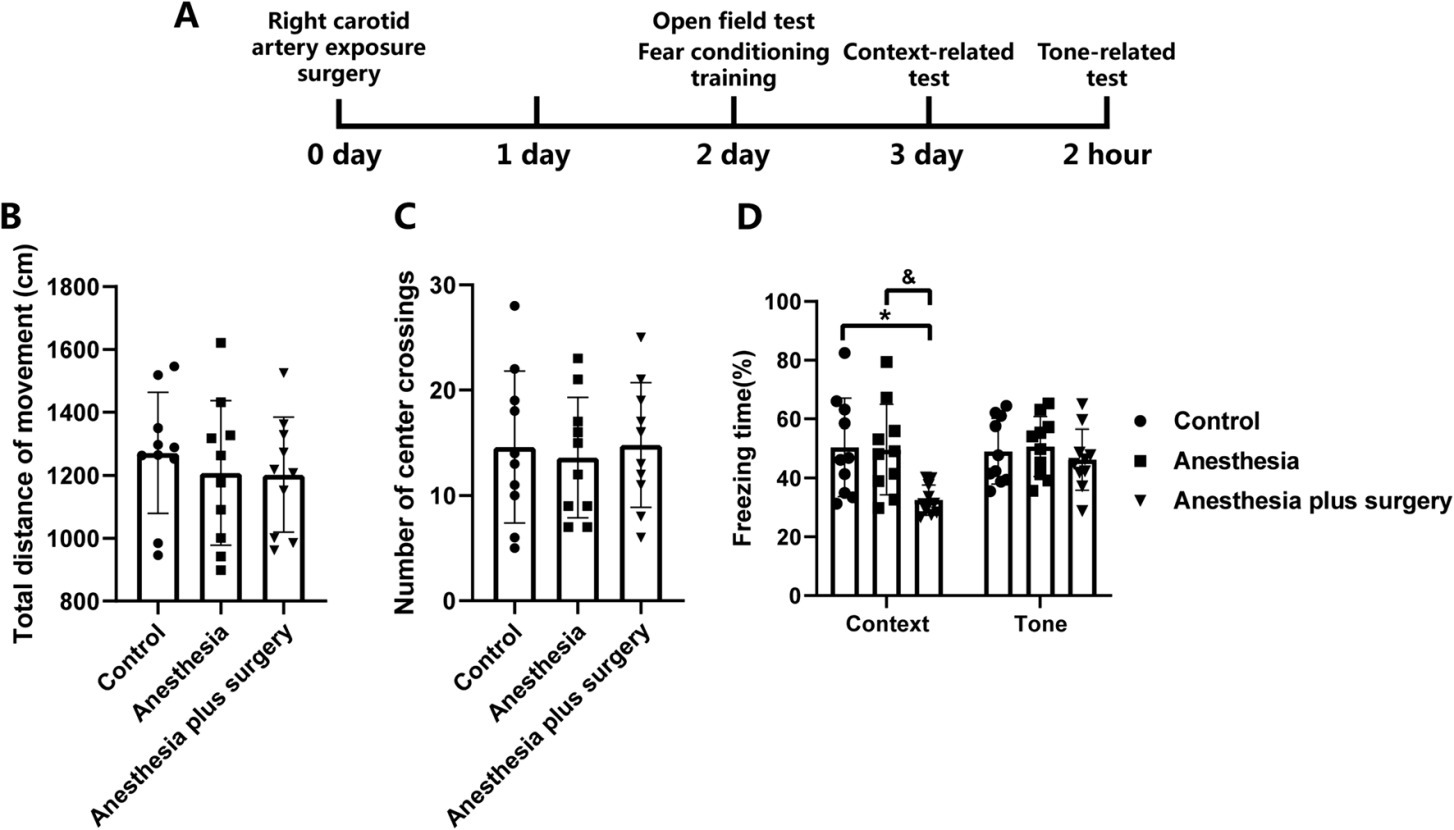
Surgical trauma, but not general anesthesia, induced postoperative cognitive deficit in aged mice. **A** Diagram of the experimental design for the neurocognitive assessment with the open field and fear conditioning test. Right carotid artery exposure surgery was performed on 0 day. The mice received the open field test and fear conditioning training on 2 days after anesthesia plus surgery. The context-relate test was conducted on 3 days after surgery. Two hours later, the tone-related test was then performed. **B** Total distance of movement in open field test. **C** Number of center crossings in open field test. **D** Freezing time for context-related and tone-related memory in fear conditioning test. *P < 0.05 versus control group, ^&^*P* < 0.05 versus anesthesia group by one-way ANOVA followed by Tukey post hoc test. *n* = 10. Data are represented as mean ± SD

We first determined the effect of anesthesia and surgical trauma on the postoperative cognitive function. No mice had an episode of hypoxia (pulse oximeter oxygen saturation < 90%) during surgery or anesthesia. All mice survived until the end of surgery. There were no statistically significance in the total distance of movement (*P* > 0.05, Fig. 1B) and number of center crossings (*P* > 0.05, Fig. 1C) between the control, anesthesia, and anesthesia plus surgery groups in open field test, suggesting that neither general anesthesia nor surgical trauma affected the postoperative motor ability of aged mice. Mice in the anesthesia plus surgery group had less context-related freezing time than either control or anesthesia group mice (*P* < 0.05, Fig. 1D), whereas no significant difference in the tone-related freezing time was found among the three groups at 3 d after surgery (*P* > 0.05, Fig. 1D). However, there was no difference in the context-related freezing time between the control and anesthesia groups at 3 d after surgery (*P* > 0.05, Fig. 1D), indicating that anesthesia by itself for a period comparable to the duration of the surgical procedure didn’t evoke postoperative cognitive deficits, thus we only evaluated the corresponding effects of surgical trauma in the following content of this study.

### Surgical trauma induced hippocampal microglial activation toward M1 polarization

**Fig. 2:**
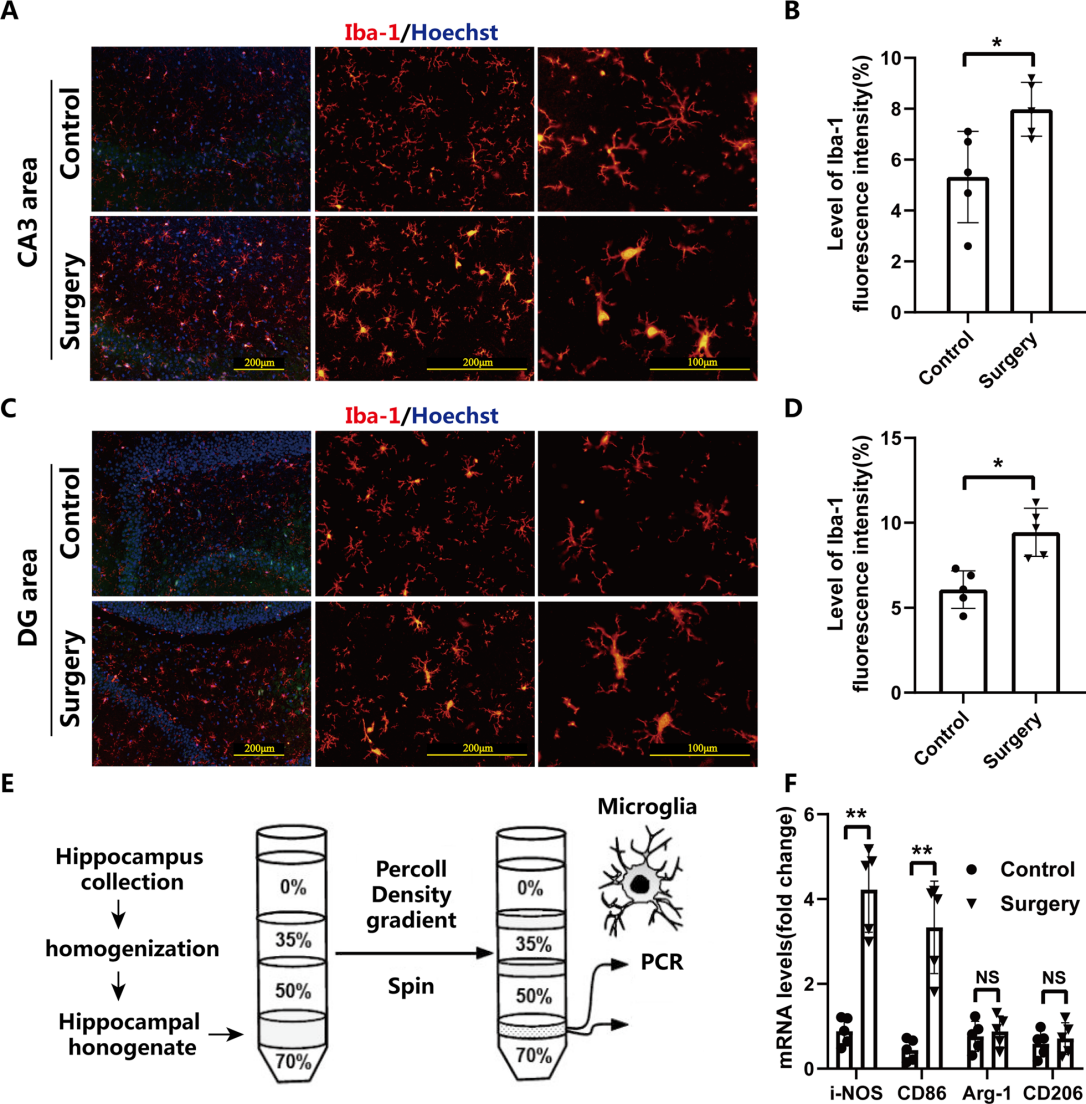
Surgical trauma induced microglial activation toward M1 polarization in hippocampus in aged mice. Representative immunofluorescence images of microglia in CA3 region (**A**) and DG region (**C**) of the hippocampus with magnification ×10 (scale bar: 200 μm) in the left panels, ×20 (scale bar: 200 μm) in the middle panels, and ×40 (scale bar: 100 μm) in the right panels, respectively; The microglia were stained with Iba-1 in red. Morphological changes of microglia were caused by surgical trauma: from "bifurcation" resting state to "short rod" or " amoeboid " activated state (**A**, **C**); Quantification of the Iba-1 fluorescence intensity in the hippocampal CA3 region (**B**) and DG region (**D**) caused by surgical trauma; **E** Microglia were separated by Percoll gradient centrifugation for RT-PCR; **F** The mRNA expression of M1(iNOS/CD86) and M2(Arg-1/CD206) phenotype markers respectively in microglia enriched by Percoll density gradient separation from hippocampus. *P < 0.05, **P < 0.01 versus control group by Student's t test. *n* = 5. Data are represented as mean ± SD

Previous studies including ours have demonstrated that postoperative cognitive dysfunction induced by surgical intervention was associated with the microglial activation [5, 22]. To further determine the phenotype of microglial activation induced by surgical trauma, we examined the activated microglial morphology and the level of Iba-1 expression in hippocampus by immunofluorescent staining. Furthermore, hippocampal microglia were collected by Percoll density gradient at 3 d after surgery to quantify the mRNA expression of its relevant M1/M2 markers. Surgical trauma induced morphological changes of microglia from "bifurcation" resting state to "short rod" or " amoeboid " activated state (Fig. 2A, C). The level of Iba-1 fluorescence intensity in the hippocampal CA3 and DG region were significantly increased in surgery group than that in control group (*P* < 0.05, Fig. 2B, D). Moreover, the results of RT-PCR showed that the expression of iNOS/CD86 (M1 marker) in enriched microglia by Percoll density gradient centrifugation (Fig. 2E) from hippocampus at 3 d after surgery was significantly higher than that in control group (*P* < 0.05, Fig. 2F), whereas the expression of Arg-1/CD206 (M2 marker) was not changed (*P* > 0.05, Fig. 2F). Collectively, these data demonstrated that surgical trauma resulted in microglial activation toward M1 polarization phenotype in the hippocampus.

### Surgical trauma increased the glucose metabolism in hippocampal region in aged mice

**Fig. 3:**
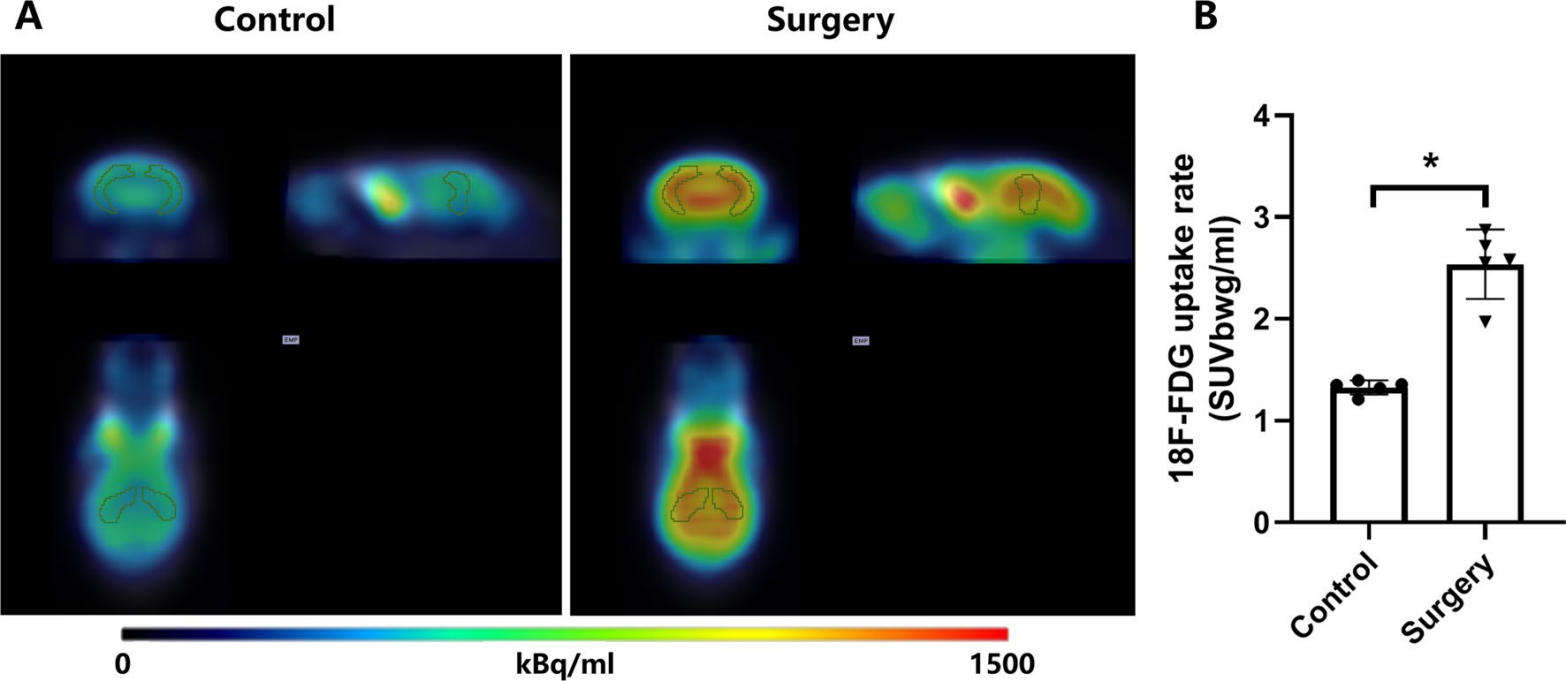
Surgical trauma increased the glucose metabolism identified by 18F-FDG PET/CT in hippocampal region. **A** Representative images of 18F-FDG PET/CT scanning in hippocampal region at 3 d after surgery. **B** Changes in 18F-FDG uptake rate of hippocampal region at 3 d after surgery. *P < 0.05 versus control group by Student's t test. *n* = 5. Data are represented as mean ± SD. The calibration bar represents the radioactivity of 18F-FDG uptake measured per unit volume (kBq/ml) with blue = 0 and red = 1500. The 18F-FDG uptake rate were standardized to body weight with SUVbwg/ml

Given that surgical trauma could mainly impair the hippocampus-dependent learning and memory in aged mice, we then utilized the 18F-FDG PET/CT, which is an effective approach in detecting cerebral glucose metabolism-related damage in neurodegenerative dementia [24, 35], to analyze the glucose metabolism in hippocampal region at 3 d after surgery. The uptake rate of 18F-FDG is a quantitative indicator that functionally reflects the glucose metabolic level of brain cells [36]. Compared with the control group, the 18F-FDG uptake rate of hippocampal area in the surgery group mice was significantly increased (Fig. 3A and B, *P* < 0.05) at 3 d after surgery, indicating that surgical trauma induced enhanced postoperative glucose metabolism in hippocampus.

### Metabolomic analysis of hippocampal tissue after surgery in aged mice

**Fig. 4:**
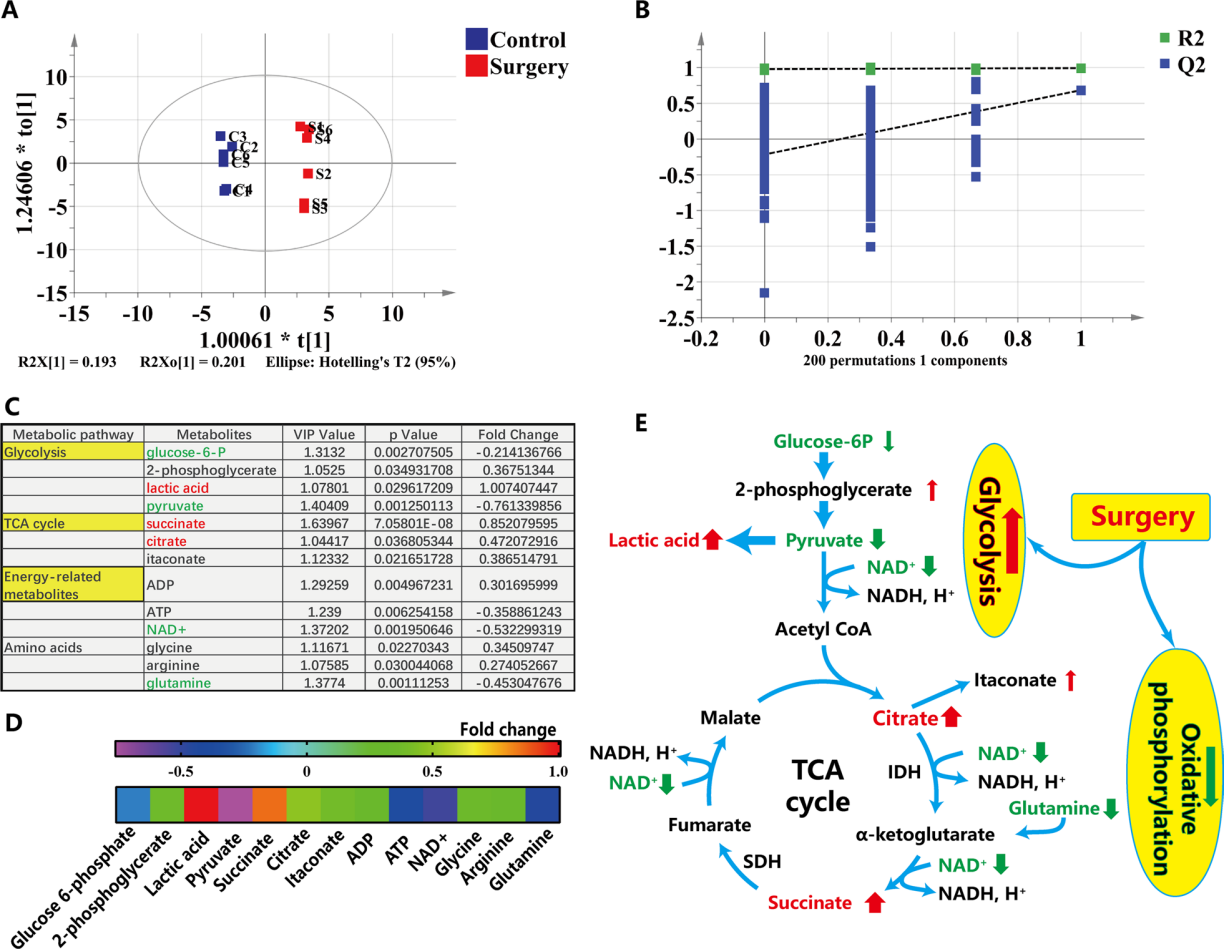
Metabolomic profiling of hippocampal tissue on basis of GC/TOF–MS technology at 3 d after surgery in aged mice. **A** OPLS-DA score plots between surgery and control group (*R*^2^Xcum = 0.68, *R*^2^Ycum = 0.993, *Q*^2^cum = 0.685); Classification showed a clear separation between the two groups; **B** Validation plots for OPLS-DA model with 200 permutation tests, intercepts: *R*^2^ = (0.0, 0.979), *Q*^2^ = (0.0, −0.215); **C** Identification of significantly differential endogenous metabolites in hippocampal tissue based on GC/TOF–MS technology; **D** Heat map reflecting the changing trend of metabolites expressed as fold-change between surgery versus control group. Ranges are indicated in red and blue (up- and downregulation in hippocampal tissue, respectively); **E** Possible metabolic pathway maps depicts the metabolic changes induced by the surgical trauma. The up arrows represent significantly increased metabolites, while down arrows represent significantly decreased metabolites. VIP indicates variables importance in the projection; OPLS-DA indicates orthogonal partial least squares discriminant analysis; *P*, significance of the pair *t*-test between surgery and control group; Fold change, ratio of relative content of metabolite in the surgery group to that of control group; TCA, tricarboxylic acid cycle

To ascertain the potential mechanism by which surgical trauma induced hippocampus-dependent postoperative cognitive deficit and glucose hypermetabolic effect, metabolomic profiles of hippocampal tissue were assessed by GC/TOF–MS technology. The score plots of OPLS-DA obtained from GC/TOF–MS data showed that the surgery and control group could be clearly differentiated with statistical significance (Fig. 4A) with the *R*^2^Ycum (0.993) and *Q*^2^cum (0.685) values exceeding 0.5, indicating that this OPLS-DA model fits the data very well and has good predictive ability. In the permutation test, all permuted *R*^2^s and *Q*^2^s are lower than the original values on the right, suggesting that the OPLS-DA model was not random or overfitted (Fig. 4B). As a result, 13 metabolites were found to be significantly different between surgery and control groups, as shown in Fig. 4C. The levels of 8 metabolites, 2-phosphoglycerate, lactic acid, succinate, citrate, itaconate, ADP, glycine, and arginine were increased significantly, while the levels of others, glucose-6-P, pyruvate, ATP, NAD^+^, and glutamine were decreased in surgery versus control group (Fig. 4C, D). Surgical trauma induced the increase of lactic acid and succinate and the decrease of pyruvate in hippocampus (Fig. 4C, D). The increase of lactic acid and decrease of pyruvate indicated the enhancement of glycolysis, whereas the accumulation of succinate, an intermediate metabolite of tricarboxylic acid cycle, indicated that the function of oxidative phosphorylation was blocked (Fig. 4C, D). Further analysis of metabolic pathways showed that glycolysis was enhanced and the function of oxidative phosphorylation related metabolic pathway was weakened (Fig. 4E). Overall, above results suggested that surgical trauma resulted in the metabolic reprogramming from oxidative phosphorylation to glycolysis in hippocampus.

### Surgical trauma-induced microglial M1 polarization and neuroinflammation in hippocampus were attenuated by glycolytic inhibitor 2-DG via inhibiting glucose metabolic reprogramming in aged mice

**Fig. 5:**
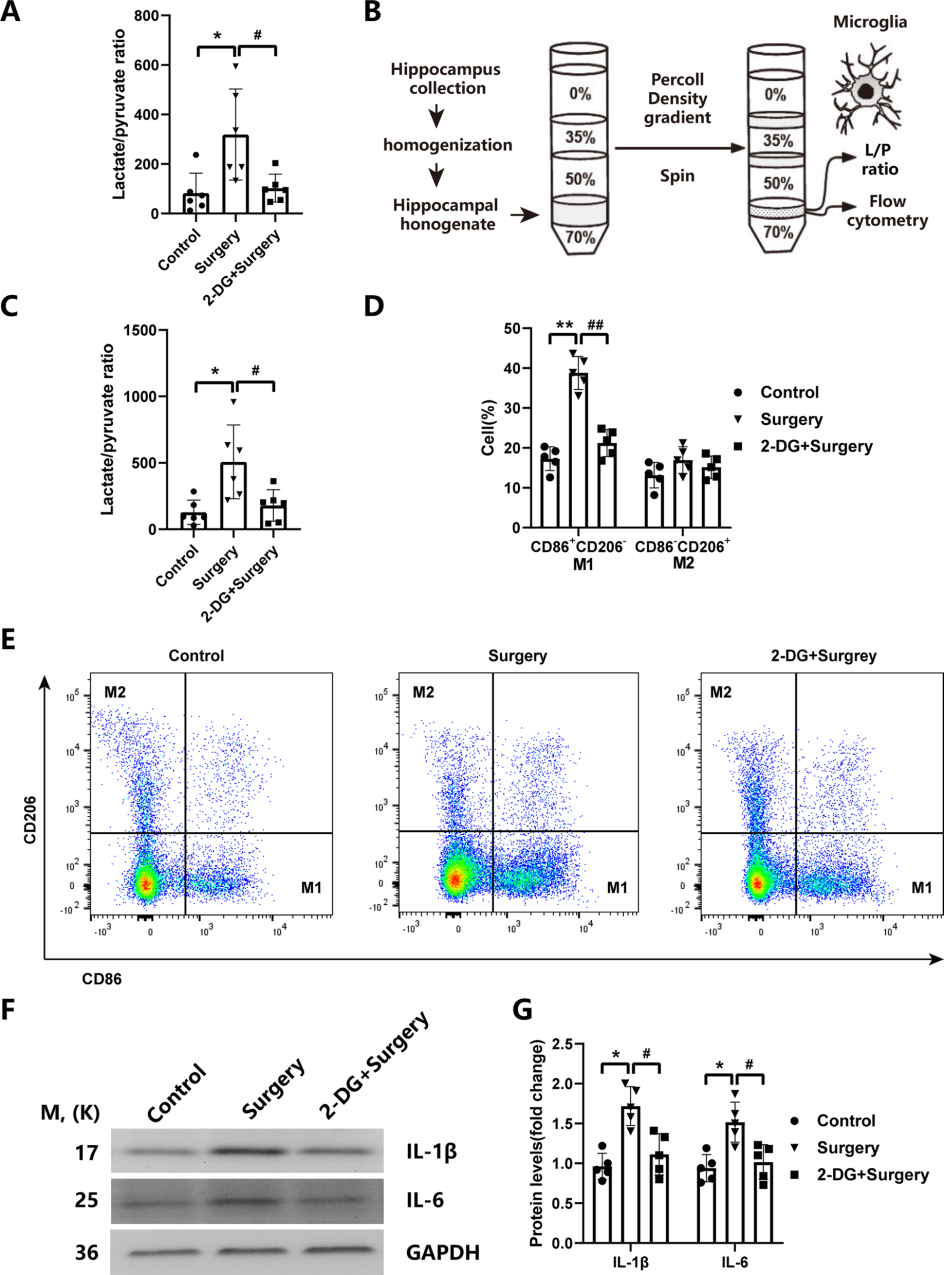
Surgical trauma-induced microglial M1 polarization and neuroinflammation in hippocampus were attenuated by 2-DG via glycolytic inhibition in aged mice. **A** The lactate/pyruvate ratio (L/P ratio) in hippocampus was examined by a Colorimetric Assay Kit; **B** Microglia were separated by Percoll gradient centrifugation for L/P ratio measurement and flow cytometric analysis; **C** The lactate/pyruvate ratio (L/P ratio) in enriched microglia was examined by a Colorimetric Assay Kit; **D** Quantification of the percentage of M1(CD86^+^CD206^−^) and M2(CD86^−^CD206^+^) phenotype in CD45^+^CD11b^+^ microglia enriched by Percoll gradient separation at 3 d after the surgery; **E** Representative dot plots showing the microglial phenotype of either M1 or M2 polarization examined by flow cytometry; **F** Western blotting images representing the expression of IL-1β and IL-6 in hippocampus; **G** Quantification for the protein levels of IL-1β and IL-6 in hippocampus at 3 d after the surgery. *P < 0.05, **P < 0.01 versus control group; ^#^*P* < 0.05, ^##^*P* < 0.01 versus surgery group by one-way ANOVA followed by Tukey post hoc test. *n* = 5. Data are represented as mean ± SD

Since our above results suggested that surgical trauma induced glucose metabolic reprogramming and microglial M1 polarization in hippocampus, we sought to further verify whether surgical trauma-induced neuroinflammation was mediated through metabolic regulation of microglial M1 polarization. We investigated the effect of the inhibition of surgical trauma induced glycolysis enhancement by 2-DG on the microglial M1 polarization and inflammatory response in hippocampus. We found that the lactate/pyruvate ratio (L/P ratio) in hippocampal tissue and enriched microglia from hippocampus was significantly increased in the surgery group compared with the control group, while it was significantly decreased in the surgery plus 2-DG group compared with the surgery group (*P* < 0.05, Fig. 5A), suggesting that 2-DG treatment abolished the surgical trauma-induced glycolysis enhancement in hippocampus. In addition, we quantified microglia subpopulations by flow cytometry in hippocampus. On the basis of CD86 and CD206 expression patterns, the microglia were classified as either M1 (pro-inflammatory) or M2 (anti-inflammatory) phenotype through flow cytometry. Flow cytometric analysis in microglia enriched by Percoll gradient separation from hippocampus revealed that the population of M1 (CD86^+^CD206^−^) was substantially increased in the surgery group comparing to control group, while this effect was significantly attenuated by 2-DG treatment (*P* < 0.05, Fig. 5B, C). Notably, no significant difference in the percentage of M2(CD86^−^CD206^+^) phenotype was found among the three groups (*P* > 0.05; Fig. 5B, C). These results indicated that the glycolytic inhibition by 2-DG alleviated the surgical trauma-induced microglial polarization toward M1 phenotype in hippocampus. Moreover, western blot assay indicated that the expression of IL-1β and IL-6 in hippocampus was markedly increased in the surgery group compared with control group, whereas the expression of these two pro-inflammatory mediators was markedly reduced in the surgery plus 2-DG group when compared with the surgery group (*P* < 0.05, Fig. 5D, E). Taken together, these data indicated that the glucose metabolic reprogramming was involved in the regulation of surgical trauma-induced microglial M1 polarization and neuroinflammation in hippocampus.

### Surgical trauma-induced postoperative cognitive decline was alleviated by glycolytic inhibitor 2-DG

**Fig. 6:**
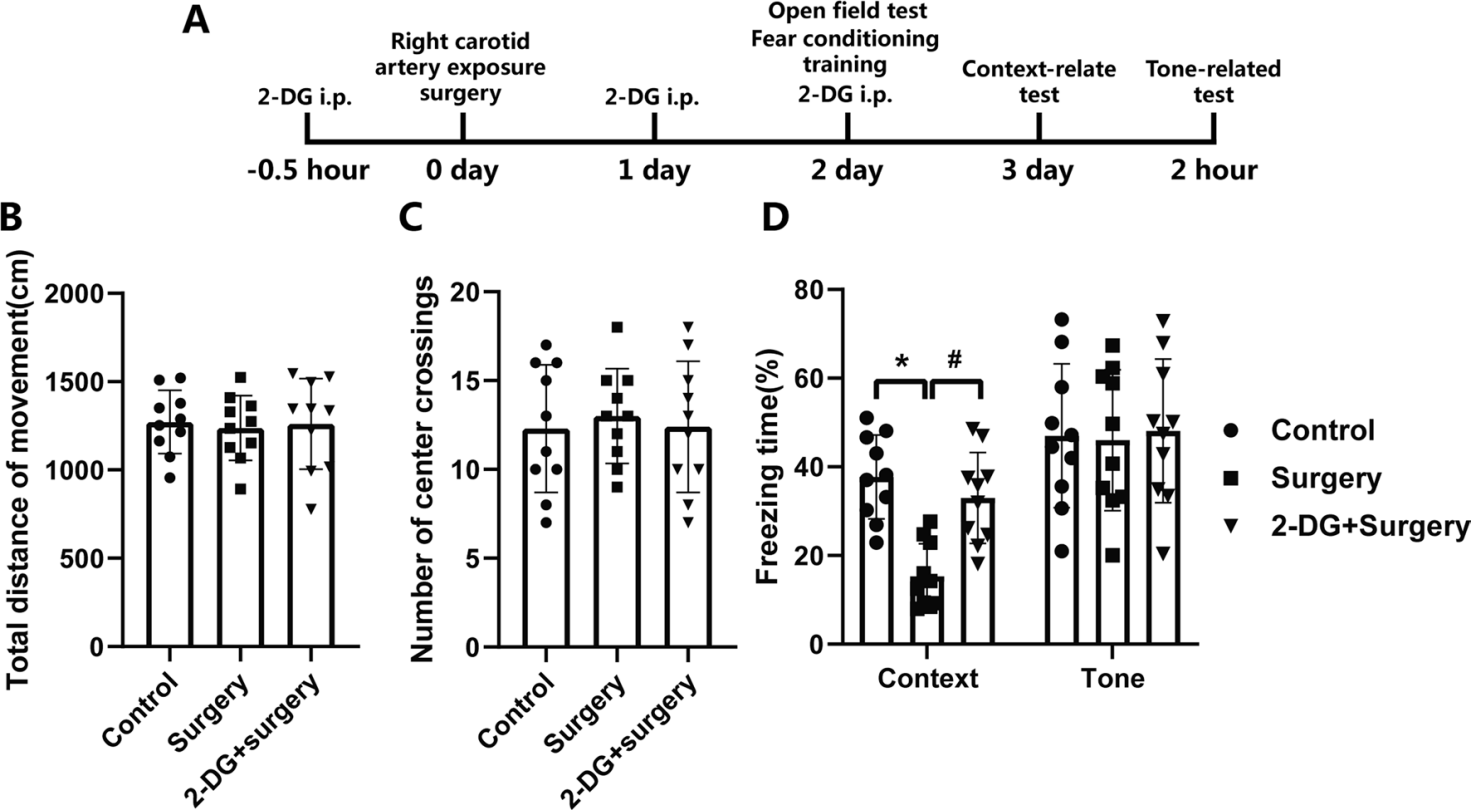
Glycolytic inhibitor 2-DG alleviated the postoperative cognitive decline induced by surgical trauma. **A** Diagram of the experimental design for the neurocognitive assessment with the open field and fear conditioning test. Right carotid artery exposure surgery was performed on 0 day. The mice received the open field test and fear conditioning training on 2 days after anesthesia plus surgery. The context-relate test was conducted on 3 days after surgery. Two hours later, the tone-related test was then performed. 2-DG was injected i.p. with 250 mg/kg/day at 0.5 h prior to surgery and then once daily on 1 day and 2 days after anesthesia plus surgery. **B** Total distance of movement in open field test. **C** Number of center crossings in open field test. **D** Freezing time for context-related and tone-related memory in fear conditioning test. *P < 0.05 versus control group, ^#^*P* < 0.05 versus surgery group by one-way ANOVA followed by Tukey post hoc test. *n* = 10. Data are represented as mean ± SD

2-DG treatment didn’t influence the total distance of movement (*P* > 0.05, Fig. 6B) and number of center crossings (*P* > 0.05, Fig. 6C) in open field test between control, surgery, and 2-DG plus surgery groups, suggesting that the postoperative spontaneous locomotor activity was not impaired by either 2-DG treatment or surgery. Mice in the surgery group had decreased freezing time for context-related memory in fear conditioning when compared with control group mice (*P* < 0.05, Fig. 6D). This decrease was attenuated by 2-DG treatment (*P* < 0.05, Fig. 6D). However, the freezing time for tone-related memory in fear conditioning was not affected by any experimental conditions (*P* > 0.05, Fig. 6D). Collectively, these results indicated that the glycolytic inhibition by 2-DG alleviated the postoperative cognitive dysfunction induced by surgical trauma.

## Discussion

The present study explored the correlation between metabolic reprogramming mediated hippocampal microglial M1 polarization and neurocognitive deficits in aged mice model of PNDs. Our data showed that surgical trauma caused a metabolic reprogramming to increase the rate of glycolysis, induced the microglial polarization toward M1 phenotype and up-regulation of inflammatory mediators in hippocampus followed by the hippocampus-dependent cognitive deficits. Additionally, inhibition of metabolic reprogramming by administration of glycolytic inhibitor (2-DG) alleviated the surgical trauma-induced microglial M1 polarization, neuroinflammation, as well as cognitive dysfunction. Together, our results suggest that metabolic reprogramming mediates hippocampal microglial M1 polarization in response to surgical trauma causing PNDs.

The evidence at hand emphasized that advanced age is the most important risk factor for PNDs [19, 25]. We have previously developed an animal model of PNDs after right carotid artery exposure surgery [22, 23, 37] which closely replicates clinical scenarios. Thus, we used this model with the aged mice to simulate the clinical status of patients with PNDs in this study. The results of the open field test indicated that the differences in total distance of movement and number of center crossings were not statistically significant, so the aged mice recovered at 2 days after surgery without development of locomotor activity decline. The mice in surgery group displayed less freezing time than the mice in control group in the context-related memory test, whereas no significant difference in the tone-related memory test was found between the control and surgery groups. Concerning the fact that context-related and tone-related freezing time in fear conditioning test reflect the hippocampus-dependent and hippocampus-independent memory respectively [38], our results demonstrated that surgical trauma mainly impaired the hippocampus-dependent memory in aged mice, which was consistent with the previous studies [22, 25, 39].

There is a growing body of evidence supporting a causal role of microglial activation and subsequent neuroinflammation in the development of PNDs [5, 22, 40, 41]. Microglia adopt different activation status toward M1 or M2 polarization phenotype [10], although the latest evidences suggests that there are intermediates states between these polar phenotypes, rather than a strict dichotomy between M1 and M2 [42]. It has been proved that the transformation of microglial polarization is in a dynamic process and the balance of M1/M2 phenotypes may be a determinant for inflammatory progression [43]. By using the ionized calcium binding adaptor molecule 1 (Iba-1), a specific protein marker expressed on the surface of microglia, we found that microglia in hippocampus after surgery exhibited the activated morphological changes, which was accompanied by increased level of Iba-1 fluorescence intensity in hippocampal CA3 and DG region. In agreement with the immunofluorescent staining profile, the expression of M1 markers (iNOS/CD86) were significantly increased in enriched microglia by Percoll density gradient centrifugation from hippocampus after surgery, whereas the expression of M2 markers (Arg-1/CD206) was not altered. These data suggest that surgical trauma caused microglial polarization toward M1 phenotype in hippocampus. Recent studies have shown that the microglial M1/M2 polarization states can be regulated by their cellular metabolism [13, 44]. In neuroinflammatory conditions, microglia undergo metabolic reprogramming, where their main metabolic pathway switches away from oxidative phosphorylation to aerobic glycolysis [10, 13]. The 18F-FDG PET/CT scanning is an effective approach in measuring cerebral glucose metabolism to elucidate potential brain-behavior relationships in animal models of human disease [45]. It has been shown that some kinds of brain insults result in cerebral glucose hypermetabolism [46, 47]. In the present study, we observed that the 18F-FDG uptake rate of hippocampal region in the surgery group was significantly increased when compared with the control group, indicating that surgical trauma induced postoperative glucose hypermetabolism in hippocampus. Moreover, we utilized GC/TOF–MS technology based metabolomic analysis to find out the potential mechanism of surgical trauma induced glucose hypermetabolic effect. We demonstrated here that surgical trauma provoked the metabolic reprogramming from oxidative phosphorylation to glycolysis in hippocampus. Taken together, our analysis revealed close temporal and spatial correlations between surgical trauma induced hippocampus-dependent cognitive impairment, microglial M1 polarization, and metabolic reprogramming in aged mice. Glucose metabolic reprogramming is a necessary step for regulation of microglial M1 polarization and subsequent pathological neuroinflammatory responses [13, 48]. A recent study shows that glycolytic inhibition by 2-DG decreased the production of LPS-induced pro-inflammatory cytokines and restored lipopolysaccharide(LPS)-induced suppression of oxidative phosphorylation in primary cultured microglia [44]. Lactate and pyruvate are both derived from glucose and therefore their extracellular concentrations partially reflect glycolytic activity. The relative proportion of lactate to pyruvate indicates the degree that glycolytically generated pyruvate is subsequently metabolized oxidatively in the mitochondria or anaerobically in the cytosol to lactate, so a higher lactate/pyruvate ratio usually represents a greater proportion of glycolysis to oxidative phosphorylation [49, 50]. In current study, we found that surgical trauma induced hippocampal glycolysis enhancement, as shown by the significantly increased lactate/pyruvate ratio in the hippocampal tissue and hippocampal microglia. Our metabolomic analysis of hippocampal tissue further corroborated the evidence of altered mitochondrial oxidative metabolism and indicated a switch to glycolytic metabolism after surgical trauma. Furthermore, flow cytometric analysis in microglia enriched by Percoll gradient separation from hippocampus revealed that the percentage of M1(CD86^+^CD206^−^) microglia were increased after surgery in aged mice, while the percentage of M2(CD86^−^CD206^+^) microglia was not changed, suggesting the microglial polarization toward M1 phenotype. Notably, 2-DG treatment abolished the surgical trauma-induced glycolytic enhancement, microglial M1 polarization, pro-inflammatory responses in hippocampus, and reversed the hippocampus-dependent postoperative cognitive dysfunction. Therefore, these data in our study support the concept that the metabolic reprogramming was involved in the regulation of surgical trauma-induced microglial M1 polarization, neuroinflammation, and neurocognitive deficits. Zhang et al. reported that sugical trauma markedly reduced the level of synaptophysin, a synaptic protein correlated with cognitive function, in aged mice [19]. Another study by York et al. demonstrated that LPS-induced glycolysis in microglia was necessary for the production of the pro-inflammatory cytokines, IL-1β, which inhibited the formation of synaptic long-term potentiation (LTP) following high frequency stimulation, whereas the addition of 2-DG to block the microglial glycolysis inhibited IL-1β production, and therefore rescued LTP in LPS-stimulated hippocampal slices [51]. The findings of the above two studies might imply that the importance of metabolic reprogramming in regulating microglial polarization with appreciable influence on pro-inflammatory cytokines release and neuronal activity. Future studies are warranted to determine what mechanism are involved in relaying the surgical trauma to metabolic reprogramming and microglial M1 polarization.

This study has several limitations that must be pointed out. First, the current study did not directly measure the metabonomics of enriched microglia by Percoll density gradient centrifugation from hippocampus due to the minimal amount of microglial specimens. Thus, it is possible that some other cells such as neurons or astrocytes might be also involved in the metabolic reprogramming effect induced by surgical trauma. However, the effect of 2-DG intervention on the L/P ratio in enriched microglia from hippocampus could, at least in part, confirm the role of the metabolic reprogramming in mediating hippocampal microglial M1 polarization and neuroinflammation. Second, microglia polarization is a dynamic process and can be altered depending upon the posttraumatic stage and its severity [52]. Microglial M1 polarization in the early stage promotes the inflammatory response, and then transition to M2 polarization boosts the recovery. We only observed that surgical trauma substantially induced hippocampal microglial M1 polarization and neuroinflammation in the pathological process of early postoperative stage, whereas microglial M2 phenotype was not changed. Nevertheless, whether the state of microglial M1/M2 polarization will be transformed at the late stage after surgery needs to be further studied. Third, we inhibited glycolysis by 2-DG to examine the regulatory effect of metabolic reprogramming on hippocampal microglial M1 polarization. In addition to glycolysis inhibition, however, the other possible functions of 2-DG [53], such as competitively inhibiting glucose transport, increasing circulation glucose levels, inducing apoptosis or autophagy, and neuroprotective effects, et al. warrant further investigation in PNDs. At last, we merely investigated the hippocampal microglial polarization after the surgery since the hippocampus-dependent postoperative cognitive dysfunction was mainly observed in our study. Indeed, investigating metabolic reprogramming mediated microglia polarization in other cognition-related brain regions besides hippocampus in response to surgical trauma may be helpful for overall assessment of the relationship between microglial M1 polarization and PNDs.

## Conclusions

In summary, our findings suggested that surgical trauma can elicit neuroinflammation and cognitive deficits, which is at least partially attributed to hippocampal microglial polarization toward M1 phenotype via glucose metabolic reprogramming. Inhibition of metabolic reprogramming represents a valuable approach to modulate microglial phenotype and thereby improve surgical trauma-induced hippocampus dependent cognitive dysfunction.

## Data Availability

The data supporting the findings of this study are presented within the manuscript.

## Abbreviations

PNDs: Perioperative neurocognitive disorders
CNS: Central nervous system
2-DG: 2-Deoxy-d-glucose
Iba-1: Ionized calcium binding adaptor molecule 1
iNOS: Inducible NO synthase
Arg-1: Arginase-1
18F-FDG: 18F-fluorodeoxyglucose
PET/CT: Positron emission tomography/computed tomography
GC/TOF–MS: Gas chromatography time-of-flight mass spectrometry
PCA: Principal component analysis
OPLS-DA: Orthogonal partial least squares discriminant analysis
VIP: Variable importance in projection
KEGG: Kyoto Encyclopedia of Genes and Genomes
GAPDH: Glyceraldehyde 3-phosphate dehydrogenase
IL-6: Interleukin-6
IL-1β: Interleukin-1β
LPS: Lipopolysaccharide
LTP: Long-term potentiation
